# Treatment of Axillary Osmidrosis by Rebalancing Skin Microecology With *Lactobacillus bulgaricus*

**DOI:** 10.3389/fmicb.2022.821696

**Published:** 2022-04-14

**Authors:** Pinglu Li, Shuyue Chen, Ping Li, Dan Xu, Xueyuan Tang, Junlin Liao, Hongju Xie, Gaofeng Li, Yehong Kuang, Juan Su, Shijie Tang, Jianda Zhou

**Affiliations:** ^1^Department of Plastic Surgery, The Third Xiangya Hospital, Central South University, Changsha, China; ^2^Department of Oncology, Third Xiangya Hospital, Central South University, Changsha, China; ^3^Department of Medical Cosmetology, The First Affiliated Hospital, University of South China, Hengyang, China; ^4^Department of Plastic and Laser Cosmetic, Hunan Provincial People's Hospital, Changsha, China; ^5^Department of Dermatology, Xiangya Hospital, Central South University, Changsha, China; ^6^Cleft Lip and Palate Treatment Center, Second Affiliated Hospital, Shantou University Medical College, Shantou, China

**Keywords:** axillary osmidrosis, *Lactobacillus*, microecological, probiotics, treatment

## Abstract

*Corynebacterium* accounts for around 20% of the armpit microbiome and plays an essential role in axillary osmidrosis (AO). In this study, the effects of *Lactobacillus bulgaricus* treatment on the microecological environment of armpits and its efficacy in the treatment of AO were investigated. A total of 10 AO patients were enrolled in this study. The patients were treated with *L. bulgaricus* mixed with saline on the left armpit (experimental group) and pure saline on the right armpit (control group) for 28 days. After treatment, AO severity showed a significant decrease (*p* = 0.013) in the experimental group compared with the control group, and the *Corynebacterium* abundance also showed a corresponding significant decrease (*p* < 0.01). Moreover, no significant variation in *Staphylococcus* abundance was found between these two groups. The microbe diversity is not disturbed in the treatment. Accordingly, our study demonstrates that *L. bulgaricus* can serve as an effective probiotic microbe for AO treatment by reducing the abundance of *Corynebacterium* and rebalancing the microecological environment.

## 1. Introduction

Axillary osmidrosis (AO), commonly known as bromhidrosis, is characterized by irritant odor emitted from the armpits. AO is ubiquitous in young people after exercise (Toyoda et al., 2016; Shin et al., 2017). The incidence of AO is unclear (Zhao et al., 2016; Chen W. et al., 2021). In our encounter survey of more than 300 college students conducted by the authors, no <30% stated that they could smell a pungent odor on both sides of their armpits after 30 min of exercise. Therefore, the prevalence of AO in young people is higher than has been reported in previous studies (Callewaert et al., 2017; Toyoda et al., 2017; Morioka et al., 2020).

Although bromhidrosis has no direct impact on physical health, it can affect the patient's social life, and some will face a certain psychological pressure because of this condition (Ozeki and Moro, 2016). Between 20 and 33% of persons with AO report that their activities of daily living are affected. Thus, the research of bromhidrosis treatment is essential.

At present, the treatment of bromhidrosis is divided into non-surgery treatment and surgery treatment (Natsch, 2015). In non-surgery treatment, drugs are used to suppress the smell or kill all associated bacteria. Whereas surgery treatment is eradication therapy, including open or laser surgery; however, pain and complications are often unavoidable, making it a hard choice for patients (Asilian et al., 2018). Finding a safe way to improve or even cure the clinical manifestations of bromhidrosis has thus become a new direction of dermatological research.

One important pathogenesis of bromhidrosis is the interaction of axillary apocrine sweat gland secretion with bacteria. Ample evidence has shown that the odor originates from the decomposition of components secreted by the apocrine sweat glands, and caused by most *Corynebacterium* species and a few cocci; as such, it is widely believed that the corynebacterial abundance is related to the occurrence of bromhidrosis (Natsch and Emter, 2020). The skin surface is composed of a large number of microorganisms. Probiotics play an essential role in treating microbial imbalances in the gastrointestinal tract, urinary tract, oral cavity, and other micro ecosystems and have become a stable and reliable component of microecological therapy (Tseng et al., 2019); however, the impact of dysbiosis on AO remains to be determined. Recently, the 16S rDNA sequencing technique has been used to detect skin bacterial components in a high-throughput manner (Zeng et al., 2020).

We decided to study whether the employment of probiotics on the skin could change the composition and diversity of its microbiome and further improve the performance of bromhidrosis (Starkenmann et al., 2005; Callewaert et al., 2017; Fujii et al., 2021). We investigated the effects of *Lactobacillus bulgaricus* treatment on the armpit microecological environment and its efficacy for AO treatment. Efficacy was evaluated by comparing the changes in the microecological environment and AO severity at targeting sites before and after treatment using previously described methods (Sun et al., 2019).

## 2. Materials and Methods

### 2.1. Ethics Statement

This study was approved by the Medical Ethics Committee of the Third Xiangya Hospital, Central South University (protocol code R21042, 16 June 2021). Written informed consent was obtained from all volunteers.

### 2.2. Reason to Adopt Lactobacillus Bulgaricus

Among the therapeutic probiotics, lactobacillus is a good facultative anaerobic bacteria, widely used in producing various drugs and foods. Among them, *L. bulgaricus* is a bacteria that can maintain activity for a long time at room temperature, and its lyophilized powder is easy to obtain. *L. bulgaricus* has been used to treat wound healing antibacterial and anti-inflammatory experiments and has made some achievements. Therefore, we chose this bacterium as a preliminary test.

### 2.3. Experimental Volunteers and Data Collection

We recruited 10 volunteers with different degrees of AO from the college students. The recruited volunteers included five males (*n*=5, 50%) and five females (*n*=5, 50%), with an average age of 24.6 ± 1.84 years old.

The detailed **inclusion criteria** were as follows: (a) males or females aged between 20 and 30 years; (b) both doctor and volunteer can smell unpleasant odors after exercising for 30 min; (c) no present infections in the axillary skin; (d) no long-term medication history; (e) no history of surgery in ampit; and (f) no drug history within 28 days. And the detailed **exclusion criteria** were as follows: (a) any active autoimmune disease or a history of autoimmune disease; (b) pregnant women of childbearing age; and (c) infectious diseases such as colds and pneumonia.

One volunteer had used antiperspirants occasionally four weeks before the experiment, while the others did not.

We evaluated the treatment efficacy from two aspects: a qualitative aspect, namely the assessment of the AO severity, and the quantitative aspect, namely the microbe component analysis. To assess the AO severity, we proposed a score method by evaluating the degrees of odor from armpits (Section 2.4). In addition, to characterize the microbe component, we adopted a DNA sequencing method to analyze the microbe component of armpits (Section 2.5).

The first round AO severity assessment and microbe component analysis (designated as S1) were conducted before the treatment. Then the volunteers go through a continuous 28-day-long treatment (Section 2.6). Within one day after the treatment, the second round AO severity assessment and microbe component analysis (designated as S2) were conducted (Section 2.7) by using the same methods as described in Sections 2.4 and 2.5.

### 2.4. Assessment of the AO Severity

We qualitatively assessed the AO severity of the volunteers before and after the treatment. To achieve this, we proposed a score method, for which we relied on some experience from previous researches (Park and Shin, 2001; Du et al., 2020) and improved upon them. The classification criteria of the proposed score method are described below.

1. The assessment took place in a closed examination room, and all volunteers were asked to wear only a light cotton shirt in order to expose their armpits fully;
2. The volunteers were asked to perform strenuous activities (e.g., exercising, jumping) for 30 min;
3. The doctor smelled the patient's armpits immediately, at distances of 50, 20, and 10 cm, respectively;
4. The doctor evaluated the scores, which can range from 0 (denoting the minimum level) to 3 (the maximum level), according to the following criteria:
  (a) Score 0, none. The patient does not give off any malodor at any distance;
  (b) Score 1, mild. The smell can only be smelled at a distance of 10 cm;
  (c) Score 2, moderate. The smell can be smelled at a distance of 20 cm;
  (d) Score 3, severe. The smell can be smelled at a distance of 50 cm.

Table 1 summarizes the score criteria and their definitions.

**Table 1:** Classification criteria of the proposed score method.

**Score^a^**	**Criteria**	**Distance**	**Smell^b^**
0	None	–	✗
1	Mild	10 cm	✓
2	Moderate	20 cm	✓
3	Severe	50 cm	✓

a *A score of 0 denotes the minimum level while a score of 3 denotes the maximum level;*

b *✗denotes that no odors can be smelled, while ✓denotes that odors can be smelled*.

### 2.5. Microbe Component Analysis

The microbe component analysis procedure comprises four steps: sample collection (Section 2.5.1), DNA extraction (Section 2.5.2), PCR amplification (Section 2.5.3), and Illumina sequencing (Section 2.5.4).

#### 2.5.1. Sample Collection

Immediately after the assessment of the AO severity, a swab sampling was conducted for all ten volunteers on their left and right armpits, respectively. We acquired 20 samples that collets the microbial community of volunteers in this step (designated as S1).

Armpit swabs were obtained using a sterile cotton swab (TinyGene company) with the following steps:

1. The doctor wearing rubber gloves moistened the sterile cotton swab with sterilized water;
2. The doctor used the cotton swab to gently swap for 20 times against the center of the armpit, by randomly choosing four areas, five times on each area, and making sure no contact with non-armpits skins to avoid contamination;
3. The sterile swab was cut at the notch;
4. The sterile swab was immediately immersed wholly in 1.5 ml of sterile 1x Phosphate Buffered Saline (PBS) preservation solution contained in a sterile 5 ml conical tube;
5. The tube was tightly sealed with a tuber lid before being immediately stored at −20°C, awaiting for DNA extraction to analyze the microbe component of armpits before the treatment.

#### 2.5.2. DNA Extraction

The proteinase K method was selected for microbe DNA extraction. The tubes containing swabs with PBS were injected with 500 μL 2% CTAB extraction buffer and 25 μL proteinase K. Then the tubes were thawed in a 37°C: water bath for 60 min to hydrolyze the DNA from microbe cells on the swabs.

Then we removed the swab and reserved the remaining resolution in the tube for DNA extraction. The tube containing the remaining resolution was centrifuged for 20 min at 10,000 rpm using a centrifuge (Thermo Scientific, Legend XR1). Next, 600 mL chloroform-isoamyl alcohol (24:1) was added, and the tubes were gently mixed for 1 min, followed by another centrifugation for 20 min at 10,000 rpm. Immediately after the second round of centrifugation, 600 μL of the supernatant from each tube was transferred to a fresh tube with 350 μL isopropanol at −20°C.

Samples were mixed by inversion and held at −20°C  for 60 min, followed by the third round of centrifugation for 10 min at 14,000 rpm, after which the supernatants were removed. The remaining DNA at the bottom of the tubes was washed with 1 mL of 70% ethanol, centrifuged for the fourth time at 14,000 rpm for 5 min. The ethanol was discarded, and another 500 μL 100% ethanol was added, centrifuged at 14,000 rpm for 10 min. At last, the ethanol was discarded, and the tubes were awaiting to dry in a sterile cabinet at room temperature. The DNA pellets were suspended in 50 mL TE buffer (10 mM Tris–HCl pH 7.6, 1 mM EDTA pH 7.6) with 2 mL ribonuclease (RNAse 20 mg/mL), incubated at 37°C for 1 h, and stored at −20°C (Doyle and Doyle, 1987).

#### 2.5.3. PCR Amplification

We used a two-step PCR amplification method to generate an amplified library for each sample. PCR reactions were performed on a GeneAmp ABI9700 PCR (Thermo Fisher Scientific Corp.) at the TinyGene Biotechnology Center (TinyGene Corp. Lmt., Shanghai, China).

Illumina sequencing is an Ultra-High-Throughput microbial community analysis technique in which multiple samples are mixed and sequenced simultaneously. In order to distinguish the source of each sample, a unique barcode primer was given for each sample during the PCR amplification procedure.

The interested-specific primer pair 357F (5′–ACTCCTACGGRAGGCAGCAG–3′) and 806R (5′–GGACTACHVGGGTWTCTAAT–3′) were adopted to amplify the V3-V4 region of the bacterial 16S rRNA gene. The primer overhang sequence 5′–AATGATACGGCGACCACCGAGATCTACAC–TCTTTCCCTACACGACGCTCTTCCGATCT–3′ was added to the 5′ end of the 357F specific primer mentioned above, acting as the forward sequencing adapter. Another primer overhang sequence 5′–CAAGCAGAAGACGGCATACGAGAT– –GTGACTGGAGTTCCTTGGCACCCGAGAATTCCA–3′ was added to the 3′ end of 806R rRNA specific primer as the reverse sequencing adapter.

##### 2.5.3.1. The First Amplification Step

The extracted DNA samples were diluted to a concentration of 25 ng/μL. To each of 20 wells of a PCR plate, 1 μL DNA sample, 10 μL 5X buffer, 1 μL (10 mM) dNTP, 5 μL templates, and 1 U Phusion Ultra fidelity DNA polymerase were added. 1 μL (10 μM) of each forward and reverse primer with unique barcode were also added. We additionally added ddH_2_O to reach a final volume of 50 μL.

All samples underwent PCR with an initial denaturation step at 94°C for 2 min. Then repeat the following PCR cycles for 38 times: 30 s of denaturation at 94°C, 30 s of annealing at 55°C, and extension for 90 s at 72°C. PCR was finished with a 5 min extension at 72°C and insulated at 10°C.

##### 2.5.3.2. The Second PCR Amplification Step

All PCR products were then collected with the electrophoresis method, by utilizing AxyPrepDNA, a 2% agarose E-gel recovery kit (AXYGEN, CORNING, America). The PCR products from 20 wells were then mixed at equal molar ratios.

Then 8 μL 5X buffer, 1 μL (10 mM) dNTP, 5 μL templates, and 0.8 U Phusion Ultra fidelity DNA polymerase were added. One microliters (10 μM) of each forward and reverse primer with unique barcode were also added. We additionally added ddH_2_O to reach a final volume of 40 μL.

All samples underwent PCR with an initial denaturation step at 94°C for 2 min. Then repeat the following PCR cycles for 8 times: 30 s of denaturation at 94°C, 30 s of annealing at 55°C, and extension for 30 s at 72°C. PCR was finished with a 5 min extension at 72°C and insulated at 10°C.

#### 2.5.4. Illumina Sequencing

Illumina sequencing is the key step in microbe analysis, whereas bidirectional DNA sequencing was performed to distinguish the microbe species. Sequencing of DNA was performed at the TinyGene Biotechnology Center (TinyGene, Shanghai, China) using the Illumina MiSeq platform (Illumina, San Diego, CA, United States). Note that we have introduced the barcodes. If the measured sequence does not contain a barcode tag sequence, the source of the sample cannot be determined, which may lead to errors in subsequent biological information or unclear meaning. Therefore, an original sequence is recognized as valid only when it contains a complete barcode tag sequence.

The PCR amplification products were collected by electrophoresis, using AxyPrepDNA, 2% agarose E-gel recovery kit (AXYGEN). The electrophoresis tank was kept clean, and the buffer was renewed. Cleaned, size-selected products were run on an Agilent Bioanalyzer to confirm appropriate profiles and determination of average sizes.

We used an FTC-3000TM instrument to perform real-time fluorescence quantification. According to the sequencing primer, high-quality sequencing fragments were categorized into groups and then clustered into operational taxonomic units (OTUs) with 97% similarity by using USEARCH (v7.0). The top 25 most abundant OTUs or tags above 10,000 were analyzed at the six levels of phylum, class, order, family, genus, and species, and the number of sequences of each sample at different classification levels were counted.

### 2.6. *Lactobacillus bulgaricus* Treatment

After the first round of AO severity assessment and microbe reanalysis, all volunteers go through a 28-day-long treatment with *L. bulgaricus*.

We dissolved 2 g *L. bulgaricus* lyophilized powder (Baiyibao Corporation, Hangzhou, China) in 10 mL saline solution (with a concentration value of 0.9%, Hunan Health Care Technology, Changsha, China) for its preparation as a therapeutic medication, adjusting the concentration of *L. bulgaricus* to 0.2 g/mL.

The 10 AO patients were treated with *L. bulgaricus* saline sprays on the left armpit (experimental group), and saline sprays on the right armpit (control group) for 28 days. Topical medication was applied once a day after taking a bath. The application range was about 2–3 cm under the armpit at a volume of 1 mL (about two sprays) each time, followed by air-drying for 1 min. Patients were not allowed to apply for any other topical medicines during the therapy and were asked to keep their diet light diet, i.e., forgo consuming spicy or stimulating food, change their underwear frequently, and keep the local skin area clean.

All ten patients in the study accomplished the 28-day-long treatment and revisited our clinic to evaluate its efficacy.

### 2.7. AO Severity Reassessment and Microbe Reanalysis

After the treatment, we reassessed the AO severity of the 10 patients to assess the treatment efficacy qualitatively. Moreover, microbe sampling was also retaken to acquire another 20 samples (designated as S2), which were used to analyze the microbe component of armpits after the treatment.

### 2.8. Statistical Analysis

Statistical analysis was performed using SPSS (Statistical Product and Service Solutions) 25.0 software (IBM, Armonk, NY, USA), and graphs were produced using the Origin Pro 2021b software (Origin, Northampton, Mass, USA). Differences in scores and microbe proportions between groups were assessed using paired *t*-testing. Correlation analyses were used to analyze the correlation factors. All data are represented as mean ± SEM (Standard Error of Mean) unless otherwise indicated. *p* < 0.05 was considered to indicate statistically significant differences.

## 3. Results

### 3.1. Scores of AO

In order to assess the effect of the treatment on AO, we recorded the scores of all the patients before and after the treatment.

**Figure 1:**
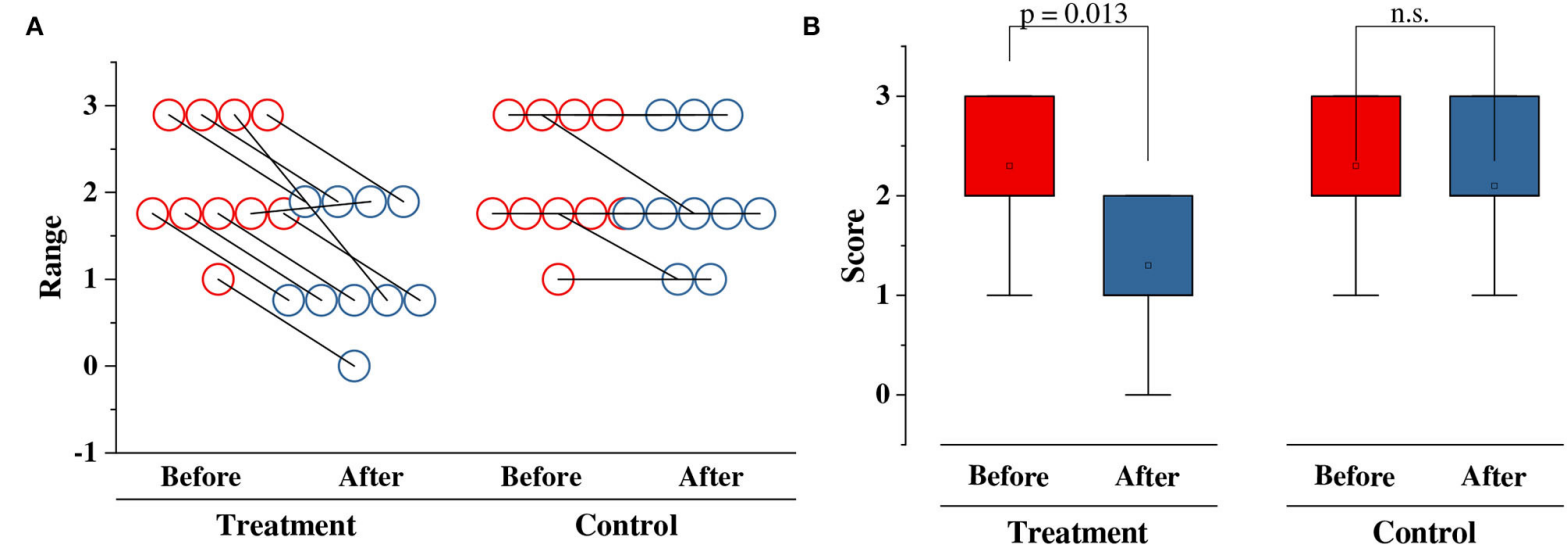
Scores in two groups: **(A)** Raw statistics; **(B)** Comparison results. n.s, no significance.

Figure 1A shows the variation in scores for the two groups. In Figure 1A, each circle represents a patient, and the scores of the same patient before and after treatment are connected by a line. In the treatment group, the scores of AO decreased in 9 out of 10 (90%) samples and only remained stable in one sample. Meanwhile, in the control group, only two samples (20%) decreased in the score.

Figure 1B further compares the statistical results of the two groups. *p*-values are calculated using a two-tailed paired *t*-test that indicates the significance of variations in the scores of the two groups. *p* < 0.05 indicates that a significant difference can be found. In the treatment group, the average score dropped from 2.3 ± 0.213 to 1.3 ± 0.213 (95% CI, *p* = 0.0013) after the 28-day treatment. The treatment group showed a significant decrease rate of 43.5%, with a confidence coefficient of 0.0013. Meanwhile, in the control group, no significant difference could be found (95% CI, *p*>0.05), although the score dropped from 2.3 ± 0.21344 to 2.1 ± 0.23333.

Therefore, *L. bulgaricus* treatment was found to significantly mitigate the degree of AO (95% CI, *p* = 0.0013).

### 3.2. Proportion of Microbe Genera

**Figure 2:**
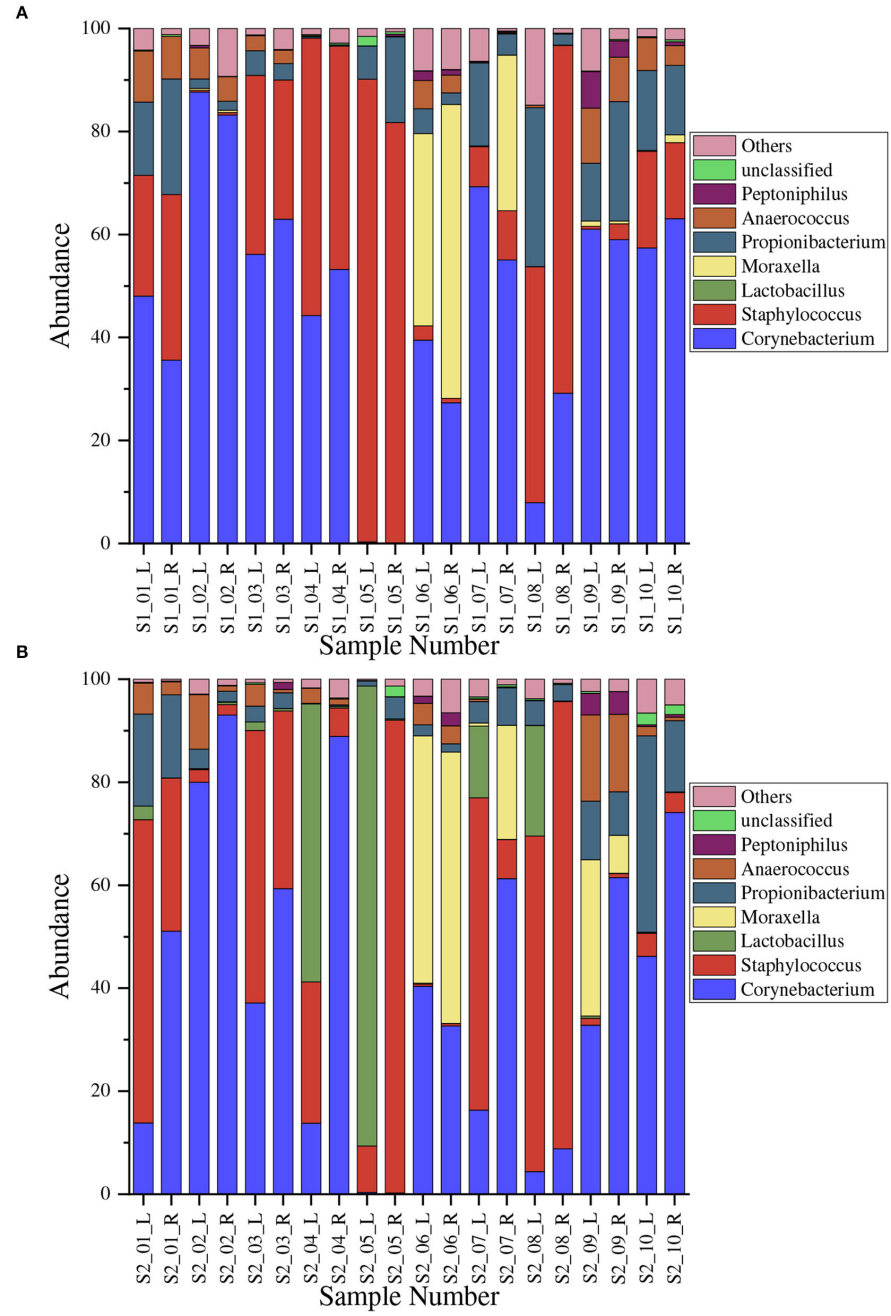
Normalized proportional distribution of microbe genera in armpits. The horizontal axis denotes different samples; for example, S1_01_L denotes the result of the left armpit of the first volunteer before the treatment. **(A)** Before the treatment (Sample S1); **(B)** After the treatment (Sample S2).

We measured the 25 most abundant OTUs in each sample, comprising a total of 22 genera that belongs to 15 major phyla.The proportions of microbes at the genera level before and after the treatment are shown in Figure 2. We only show the top seven genera; namely, *Corynebacterium, Staphylococcus, L. bulgaricus, Moraxella, Propionibacterium, Anaerococcus*, and *Peptoniphilus*. *Corynebacterium* species dominated, ranging from 0.08 to 88% in Figure 2A and 0.2 to 93% in Figure 2B, with mean values of 0.47009 ± 0.24518 and 0.40806 ± 0.29768, respectively. *Staphylococcus* was the second-most dominant microbe genus, ranging from 0.3 to 89% in Figure 2A and 0.4 to 92% in Figure 2B, with mean values of 0.27961 ± 0.27961 and 0.27287 ± 0.31028, respectively.

**Figure 3:**
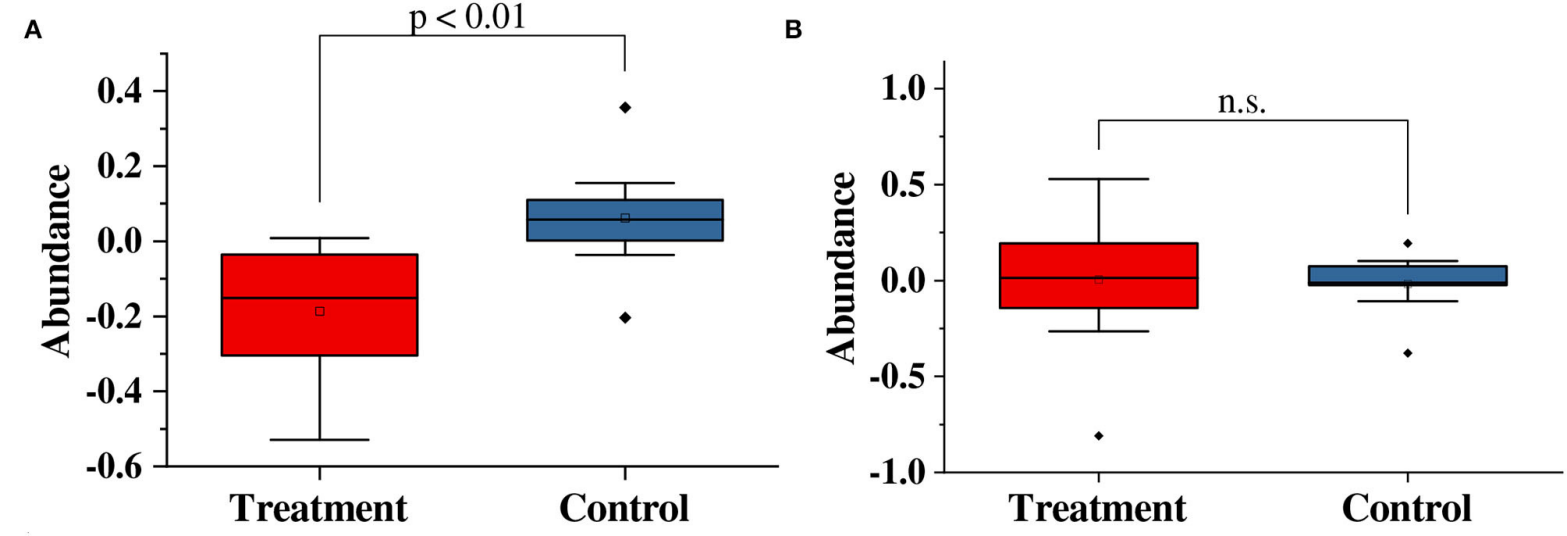
Paired *t*-test results for variations in the abundance of **(A)** *Corynebacterium* and **(B)** *Staphylococcus* in this study. n.s, no significance.

We compare the variation in genus abundance between the treatment group and the control group in Figure 3. In Figure 3A, the abundance of *Corynebacterium* was significantly decreased (*p* < 0.01) in the treatment group compared with the control group. In the treatment group, the abundance of corynebacteria dropped by 18.6 ± 5.6%; while, in the control group, the abundance of corynebacteria increased by 6.2 ± 4.5%. Moreover, the abundance of *Staphylococcus* showed no significant variation (*p*>0.05) in the treatment group compared with the control group.

### 3.3. Microbe Diversity

**Figure 4:**
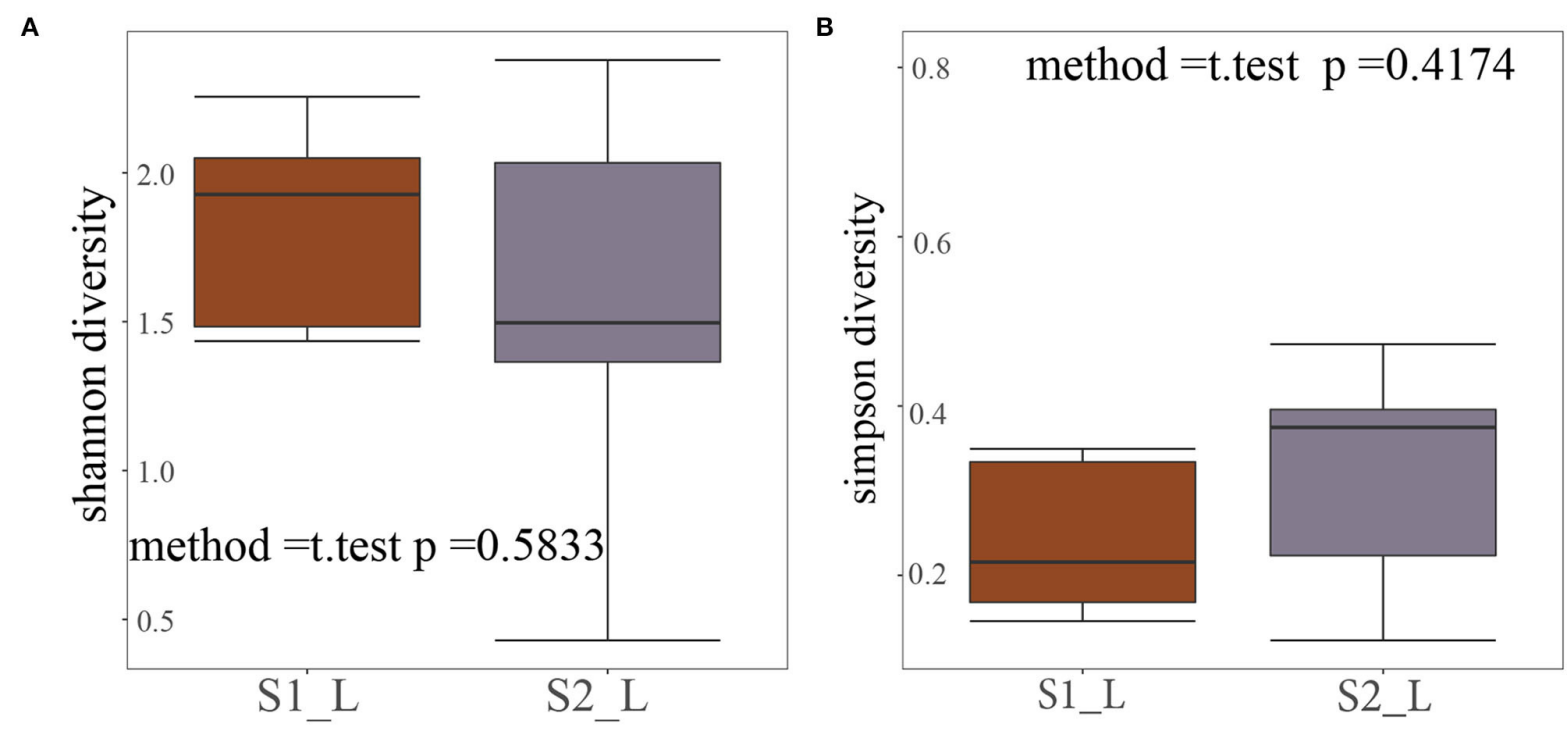
Microbe diversity. **(A)** Shannon alpha diversity. **(B)** Simpson alpha diversity.

We use Shannon and Simpson alpha diversity indicator to examine the safety of treatment. According to the T-test results in Figure 4, No significant variation in the microbe diversity before and after the treatment can be found in both indicators. More specifically, in Figure 4A, no significant change can be found in the Shannon diversity before and after the treatment (*p* = 0.5833>0.05). As for the Simpson diversity in Figure 4B, it is not significantly changed by the treatment neither (*p* = 0.4174>0.05). Thus, the microbe diversity is not destroyed by our treatment, making it a safe solution.

## 4. Discussion

AO is an unpleasant body odor or osmidrosis that occurs secondary to excessive secretion from the apocrine gland that becomes malodorous on bacterial activities (Semkova et al., 2015). In recent years, various therapeutic modalities have been developed to help address these concerns (Malik et al., 2021), including non-surgery treatment and surgery treatment (Natsch, 2015).

Non-surgery treatment is the first-line therapy where drugs are used to suppress the smell or kill all associated bacteria. Prior works of non-surgery treatment include hygiene modification and topical agents. Hygiene modification is a method to suppress the smell, including showers, laundering, antiperspirant application. However, the odor will reappear immediately by adopting hygiene modification methods, which can be annoying. For example, to eliminate the odor, patients will have to shower repeatedly and frequently, i.e., several times a day. While in topical agents, drugs like ozone oil, antibiotic and antimicrobial metal ions therapies are widely used, where all associated bacteria are killed. Thus, hygiene modification can be troublesome, while topical agents can irritate the balance of skin microbiology (Benohanian et al., 1998).

Surgery treatment is a eradication solution, including microwave and micro-needle (Li et al., 2020) surgeries, which usually adopts open or laser surgery to kill the apocrine gland. However, pain and complications are often unavoidable in surgery treatment, making it a hard choice for patients (Feldmeyer et al., 2015; Asilian et al., 2018). Moreover, these surgeries might lead to scar hyperplasia of the skin (Huang et al., 2021).

Thus, finding a safe and feasible way to improve or even cure the clinical manifestations of AO is essential in dermatological research. The skin surface has a huge collection of microbial flora. The balance of flora sustains the normal body odor. Probiotic therapy is widely used in the treatment of oral and intestinal diseases (Chen M.J. et al., 2021; Silva et al., 2021), even used in the mental and neurological diseases (Ahmad et al., 2019; Shabbir et al., 2021). In recent years, some researchers tried to figure out the possibility of probiotic treatment for axillary osmidrosis, but there has been no specific conclusion (James et al., 2013). In this study, we explored whether we could improve the balance of axillary flora by using probiotics so as to relieve axillary osmidrosis.

This paper investigated the effects of *L. bulgaricus* treatment on the microecological environment of armpits and its efficacy in the treatment of AO. Our work is a kind of non-surgery care and can provide an alternative choice for patients to alleviate AO symptoms.

We firstly investigated the effects of *L. bulgaricus* treatment on the armpit microecological environment. After treatment, the *Corynebacterium* abundance significantly decreased (*p* < 0.01) in the experimental group compared with the control group. Moreover, the two groups showed no significant variation with respect to the *Staphylococcus* abundance, indicating the results were specific for *Corynebacterium*. Moreover, unlike antibiotic methods, the microbe diversity is not destroyed, making the proposed solution feasible and safe.

We also evaluated the treatment efficacy by comparing AO severity before and after the treatment. AO severity showed a significant decrease in the experimental group compared with the control group (*p* = 0.013). Therefore, our results demonstrate that *L. bulgaricus* treatment can effectively reduce the severity of AO by decreasing the abundance of *Corynebacterium*. Other scholars (Zeng et al., 2021) have hypothesized that *Corynebacterium* may be pathogenic, but Staphylococcus may be probiotic for AO, indicating that *Corynebacterium* is a harmful bacterium and *Staphylococcus* may be a protective bacterium, which is inconsistent with our findings. The addition of *L. bulgaricu* in our experiment might lead to the insignificant increase in the abundance of *Staphylococcus*.

According to the data before and after the experiment, we can see that the bacterial abundance of volunteer No. 5 is significantly different from others. Because No. 5 used antiperspirant intermittently four weeks ago. According to the experimental results, we can find that the content of Staphylococcus in her is significantly increased, which coincides with the significant increase of Staphylococcus in patients who use antiperspirants for a long time. However, because the volunteers stopped taking drugs for some time, we speculated that the previous intermittent use of drugs had destroyed the microecology balance of the axillary skin. We realized that the use of antiperspirants and fungicides would indeed destroy the balance of microbial flora. We can tell the microbial flora of other patients had not been significantly damaged after using probiotics, so this might be a better way to treat AO. It is worth mentioning that all 10 volunteers did not emerge any discomfort, such as itching, redness, and swelling.

This trial has some limitations. As for future works, we could increase the samples and select people of different ages with or without AO to get a more significant result. Moreover, experiments on people of all ages should also be carried out to make the study more convincing. To achieve this end, we will experiment on middle-aged and older people who are troubled with AO. Once successful, the experiment will benefit those people's lives with lower costs than traditional methods.

Furthermore, we should also analyze more bacteria to find other related ones. We indicate that more types of bacteria can be tried. This study only investigated one type of bacteria, the *L. bulgaricus*. Other bacteria may also be effective. In subsequent experiments, we will continue to explore whether bifidobacteria or other bacteria can improve the performance of AO. However, due to the strict growth environment of other bacteria, making and preserving products is also a problem we need to solve.

For further study, we will make productions like emulsion, spray, perfume, and other products with *L. bulgaricus* so that consumers with AO can buy them freely. We will continue to investigate the safety and effectiveness of products under different conditions. We hope to promote the industrialization of the pharmaceutical industry and make clinical research more beneficial to people.

## 5. Conclusion

Axillary Osmidrosis (AO) is a chronic condition in which an unpleasant odor emanates from the armpits. This paper investigated the effects of *L. bulgaricus* treatment on the microecological environment of armpits and its efficacy in the treatment of AO.

AO severity showed a significant decrease in the experimental group compared with the control group (*p* = 0.013). After treatment, the *Corynebacterium* abundance significantly decreased (*p* < 0.01) in the experimental group compared with the control group; moreover, the two groups showed no significant variation with respect to the *Staphylococcus* abundance, indicating the results were specific for *Corynebacterium*.

Therefore, our results demonstrate that *L. bulgaricus* treatment can effectively reduce the severity of AO by decreasing the abundance of corynebacteria. Noticeably, unlike the antibiotic method, in our treatment, the microbe diversity is not destroyed, making the proposed solution feasible and safe.

## Data Availability Statement

The original contributions presented in the study are included in the article/supplementary materials, further inquiries can be directed to the corresponding author/s.

## Ethics Statement

The studies involving human participants were reviewed and approved by Medical Ethics Committee of the Third Xiangya Hospital of Central South University. The patients/participants provided their written informed consent to participate in this study.

## Author Contributions

PingluL: conceptualization and formal analysis. PingluL, XT, and GL: data curation. JS and JZ: funding acquisition. PingluL, DX, YK, ST, and JZ: investigation. PingluL, JL, and HX: methodology. YK, JS, and JZ: project administration. SC, PingL, DX, XT, ST, and JZ: resources. HX: software. PingL, GL, and JZ: supervision. PingluL, SC, JL, and JZ: validation. PingluL and JZ: writing original draft and writing review and editing. All authors contributed to the article and approved the submitted version.

## Funding

This research was funded by the Key Research and Development Program of Hunan Province, Science and Technology Department of Hunan Province, grant numbers 2018SK2081, 2018SK2083, and 2018SK2084; Hunan Science and Technology Innovation Plan, grant number 2018JJ2616; 2020 Li Ka Shing Foundation Cross-Disciplinary Research Grant, grant numbers 2020LKSFG18B and 2020 LKSFG02E; Project of Science and Technology of Hunan Province No. 2021JJ40932; and The Changsha Municipal Natural Science Foundation No. kq2007038.

## Conflict of Interest

The authors declare that the research was conducted in the absence of any commercial or financial relationships that could be construed as a potential conflict of interest.

## Publisher's Note

All claims expressed in this article are solely those of the authors and do not necessarily represent those of their affiliated organizations, or those of the publisher, the editors and the reviewers. Any product that may be evaluated in this article, or claim that may be made by its manufacturer, is not guaranteed or endorsed by the publisher.
